# The Prevalence of Psychological Symptoms in Pregnant Healthcare Workers (HCWs) and Pregnant Non-HCWs During the Early Stage of COVID-19 Pandemic in Chongqing, China

**DOI:** 10.3389/fpsyt.2021.708698

**Published:** 2021-08-25

**Authors:** Min Liu, Nan Li, Xianghao Cai, Xiaoyan Feng, Rong Wang, Peng Xiong

**Affiliations:** ^1^Department of Public Health and Preventive Medicine, School of Medicine, Jinan University, Guangzhou, China; ^2^Department of Obstetrics and Gynecology, The First Affiliated Hospital of Jinan University, Guangzhou, China; ^3^Department of Obstetrics, The First Affiliated Hospital of ChongQing Medical University Jinshan Hospital, Chongqing, China; ^4^Department of Intensive Care Unit, Taihe Hospital, The Affiliated Hospital of Hubei, University of Medicine, Shiyan, China

**Keywords:** COVID-19, pregnant healthcare workers, psychological symptoms, pregnant women, Chinese

## Abstract

**Background:** Studies showed that healthcare workers (HCWs) and pregnant women bore the burden of mental problems during the coronavirus disease 2019 (COVID-19) pandemic. While, few studies have focused on the psychological impact of COVID-19 pandemic on pregnant women who work at healthcare settings. This study aimed to investigate and compare the prevalence difference of psychological symptoms between pregnant HCWs and pregnant non-HCWs during the early stage of COVID-19 pandemic in China.

**Methods:** A cross-sectional online survey with anonymous structured questionnaires was conducted from February 15 to March 9, 2020. A total of 205 pregnant women in Chongqing, China were recruited. The mental health status was assessed using symptom checklist-90 (SCL-90).

**Results:** Our sample was composed of 83 pregnant HCWs (mean age = 29.8) and 122 pregnant non-HCWs (mean age = 30.8). The results suggested the prevalence of psychological symptoms (the factor score ≥2) among all pregnant women ranged from 6.83% (psychosis symptoms) to 17.56% (obsessive-compulsive symptoms). Compared with pregnant non-HCWs, pregnant HCWs reported higher prevalence of psychological symptoms in 10 factors of SCL-90. After controlling the confounding variables, multiple logistic regression demonstrated that pregnant HCWs experienced higher prevalence of psychological symptoms of somatization (18.07 vs. 5.74%, *p* = 0.006, aOR = 4.52), anxiety disorders (16.87 vs. 6.56%, *p* = 0.016, aOR = 3.54), and hostility (24.10 vs. 10.66%, *p* = 0.027, aOR = 2.70) than those among pregnant non-HCW.

**Conclusion:** Our study indicated that pregnant HCWs were more likely to suffer from mental health distress than pregnant non-HCWs during the early stage of COVID-19 pandemic. It is vital to implement targeted psychological interventions for pregnant women, especially for pregnant HCWs to cope with distress when facing the emerging infectious diseases.

## Introduction

Coronavirus disease 2019 (COVID-19) continues to spread throughout many countries and territories since it broke out in December, 2019 (1, 2). On July 15, 2021, it was reported that more than 180 million cases were confirmed worldwide and more than 4 million patients died (3). More and more evidence has indicated that the COVID-19 pandemic has seriously threatened the physical and mental health status of the public (4–8).

As a vulnerable group, pregnant women have been at a high risk of experiencing the burden of mental problems during the COVID-19 pandemic, which might be due to the fear of COVID-19 (such as the fear of infecting others or loved ones) (9), and stressful events resulting from the pandemic (such as the death of relatives, interpersonal imbalances, lack of contact with relatives, and occupational problems) (10, 11). During the early stage of the pandemic, it was reported that pregnant women had a high prevalence of psychological symptoms. Dong et al. (12), for example, investigated the mental status of Chinese pregnant women from February 22 to February 27, 2020 and reported that 8.3% had anxiety and 50.6% had depression. Zhang et al. (13) found that 40% of Chinese pregnant women (total sample = 1,901) had suspected posttraumatic stress disorder (PTSD) from February 13 to March 16, 2020. Zhang et al. (14) reported that 67.1% of pregnant women experienced moderate-to-severe psychological impact during February and March, 2020, in Liaoning, China. Mental distress during pregnancy can have adverse consequences on pregnant women and the fetus (15), which indicates that this population should receive full attention in the context of the COVID-19 pandemic.

Faced with the public health crisis, healthcare workers (HCWs) have also experienced considerable psychological distress. Studies that have focused on the psychological impact of previous infectious outbreaks, such as the severe acute respiratory syndrome (SARS) and Ebola epidemics, have found that HCWs suffered from various mental problems, including anxiety, depression, and PTSD (16, 17). Similarly, several studies have shown that HCWs experienced a high level of mental distress during the COVID-19 pandemic (18–22). A recent systematic review and meta-analysis that included 62 primary studies at the early stage of the COVID-19 pandemic summarized that the pooled prevalence of anxiety and depression among HCWs were 26 and 25%, respectively, which indicates that this population are vulnerable to mental distress (23). This might be due to the lack of human resources, long-term workload (18), the high risk of exposure to COVID-19 (18, 19), the lack of rest (24), the high infection rate among this population (24, 25), and poor social support and self-efficacy (26). Compared with general population, HCWs reported a much higher prevalence of psychological problems during the early stage of the COVID-19 pandemic in China. Xiao et al. (27), for example, reported that 54.2 and 58% of HCWs across 26 provinces in China had symptoms of anxiety and depression, respectively, after January 28, 2020, during the peak of the COVID-19 epidemic. Zhang et al. found that, compared with non-HCWs (*n* = 1,255), HCWs (*n* = 927) had a higher prevalence of insomnia (38.4 vs. 30.5%; *p* < 0.01), anxiety (13 vs. 8.5%, *p* < 0.01), depression (12.2 vs. 9.5%; *p* < 0.04), somatization (1.6 vs. 0.4%; *p* < 0.01), and obsessive-compulsive symptoms (5.3 vs. 2.2%; *p* < 0.01) from February 19 to March 6, 2020 (19). Zhou et al. also documented similar results, whereby frontline HCWs (*n* = 606) had higher levels of depressive symptoms (57.6 vs. 47.6%; *p* < 0.001), anxiety symptoms (45.4 vs. 33.8%; *p* < 0.001), somatic symptoms (12 vs. 7.7%; *p* = 0.003), and insomnia (32 vs. 25.1%; *p* = 0.002) than the general population (*n* = 1,099), from February 14 to March 29, 2020 (18). Lu et al. reported that, compared with hospital administrative staff, HCWs were 1.4 times more likely to feel fear and twice as likely to suffer from anxiety and depression between February 25 and March 26, 2020 (22).

Due to the shortage of medical human resources during the COVID-19 pandemic, early pregnant HCWs might need to stay in job, but work in the non-frontline contact and low-risk of infection units. Adding the compromised immunological functions and physiological changes that occur during pregnancy, this special work environment might increase the risk of complications in these women (28, 29). Being familiar with the occupational health policy of a hospital to seek a safe work environment in the early stage of the COVID-19 pandemic was challenging for pregnant HCWs (30, 31). In this situation, pregnant HCWs might face greater psychological pressure and more complicated psychological problems.

However, the psychological impact of COVID-19 pandemic on pregnant HCWs, relative to pregnant non-HCWs, has not been extensively assessed. Furthermore, few studies have investigated the psychological impact of the COVID-19 pandemic on pregnant HCWs in Chongqing, which is a municipality in Southwest China with a population of more than 31 million. Hence, this study investigated the prevalence of psychological symptoms, including symptoms of somatization, obsessive-compulsive disorder, interpersonal sensitivity, depression, anxiety, hostility, phobic anxiety, paranoid ideation, and psychoticism, in both pregnant HCWs and pregnant non-HCWs during the early stage of the COVID-19 pandemic in Chongqing, China. The results could aid the development of an effective intervention for controlling the emerging comprehensive psychological health issues for pregnant women, especially pregnant HCWs during the COVID-19 pandemic.

## Methods

### Participants and Procedure

A cross-sectional study was performed to assess the psychological status among pregnant women during the early stage of the COVID-19 pandemic, between February 15 and March 9, 2020. As the Chinese government encouraged the public to stay at home, subjects were electronically invited to participate by completing an anonymous online survey (*via* wjx.cn, which is a popular online survey platform in China). Women aged 18 years or older who were pregnant at the time of the survey were recruited. Women with cognitive disorders, severe mental illnesses (such as major depression, schizophrenia, and bipolar disorder) or other serious diseases diagnosed before our investigation, and those who failed to fill out the questionnaire by themselves, were excluded. Medical staffs were recruited if they met the inclusion criteria. The present study was approved by the Institutional Review Board of Jinshan Hospital, the First Affiliated Hospital of Chongqing Medical University. All participants gave signed e-written informed consent before the start of the survey. The investigation was conducted in accordance with the latest version of the Declaration of Helsinki.

### Measurements

#### Demographics

Demographic information was collected, including occupation (pregnant HCWs and pregnant non-HCWs), age, education, gestation period, mode of gestation, and number of fetuses per pregnancy. In the questionnaire, first trimester, second trimester, and third trimester referred to the gestation periods of 1–12, 13–28 weeks, and more than 28 weeks, respectively.

#### Mental Health Status

Self-reported mental health symptoms were assessed using the Symptom Checklist-90 (SCL-90). The reliability and validity of the Chinese version of the SCL-90 have been established in previous studies (32, 33). The inventory contains 90 questions that evaluate 10 primary symptom factors, including somatization, obsessive-compulsive, interpersonal sensitivity, depression, anxiety, hostility, phobic anxiety, paranoid ideation, psychoticism, and additional items (e.g., appetite and sleep) in the last week. Each of the 10 symptom factors contains 6–13 items. All items were graded on a 5-point Likert scale, ranging from “1 = not at all” to “5 = extremely,” with a higher score indicating more frequency and intensity of psychological symptoms. The mean score of each factor was used as the indicator to evaluate the mental health status. When a factor score was ≥2, it was considered the occurrence of mental health problems in that factor. In this study, the Cronbach's alpha of this scale was 0.99, which indicates a good reliability (34).

#### Statistical Analysis

Descriptive statistics were used to characterize the sample. Continuous variables are presented as the mean ± SD, whereas categorical variables are presented as cases (*n*) and percentage (%). *t*-Tests and chi-squared tests were used to examine the between-group difference in continuous variables and categorical variables, respectively. Multiple logistic regression models were fit to examine the association between psychological symptoms and occupation (pregnant HCWs and pregnant non-HCWs), adjusted for potential confounders. In addition, sensitivity analysis was performed. We further divided pregnant non-HCWs into two subgroups according to whether they were or were not working. Then, three groups were as follows: group 1: not working pregnant non-HCWs; group 2: working non-HCWs; and group 3: pregnant HCWs. Differences in prevalence of psychological symptoms between the three subgroups were further analyzed using chi-squared tests. Statistical significance was considered a two-tailed *p*-value < 0.05. All analyses were performed using Stata 14 (STATA Corp., TX, USA) (35).

## Results

### Descriptive Statistics

Of the 205 participants, the average age was 30.4 years (SD = 3.4 years). Generally, participants were highly educated (95.61% had a university degree or above). More than half of the participants (60%) were in the third trimester. Most participants got a natural pregnancy (96.59%) and had singleton pregnancy in this pregnancy (97.56%). In addition, 74.15 and 77.07% of pregnant women were afraid of infection of themselves and their fetus, respectively. Of all participants, 29.76% reported having the need for psychological counseling. Detailed information is provided in Table 1.

**Table 1:** Sociodemography between pregnant HCWs and pregnant non-HCWs.

**Variable**	***N* (%)**	**Pregnant HCWs (%)**	**Pregnant non-HCWs (%)**	***t*/χ** ^**2**^	***p*-Value**
Age [mean (SD)]	30.4 (3.4)	29.8 (2.88)	30.8 (3.78)	2.110	0.036^*^
≤30 years old	116 (56.59%)	62 (65.06%)	54 (50.82%)	4.078	0.043^*^
>30 years old	89 (43.41%)	60 (34.94%)	29 (49.18%)	–	–
*Education*
High school or less	9 (4.39%)	1 (1.20%)	8 (6.56%)	–	0.087
University degree or above	196 (95.61%)	82 (98.80%)	114 (93.44%)	–	–
*Gestation period* ^*a*^
First trimester	21 (10.24%)	20 (24.10%)	1 (0.82%)	–	<0.001^*^
Second trimester	61 (29.76%)	34 (40.96%)	27 (22.13%)	–	–
Third trimester	123 (60.00%)	29 (34.94%)	94 (77.05%)	–	–
*Mode of gestation*
Natural pregnancy	198 (96.59%)	80 (96.39%)	118 (96.72%)	–	1.000
Assisted reproductive technology	7 (3.41%)	3 (3.61%)	4 (3.28%)	–	–
*Number of pregnant fetuses*
Singleton pregnancy	200 (97.56%)	81 (97.59%)	119 (97.54%)	–	1.000
Twin pregnancy	5 (2.44%)	2 (2.41%)	3 (2.46%)	–	–
*Fear of infection*
Yes	152 (74.15%)	73 (87.95%)	79 (64.75%)	13.866	<0.001^*^
No	53 (25.85%)	10 (12.05%)	43 (35.25%)	–	–
*Fear the fetus being infected*
Yes	158 (77.07%)	74 (89.16%)	84 (68.85%)	11.524	0.001^*^
No	47 (22.93%)	9 (10.84%)	38 (31.15%)	–	–
*Need psychological counseling*
Yes	61 (29.76%)	21 (25.30%)	40 (32.79%)	1.324	0.250
No	144 (70.24%)	62 (74.70%)	82 (67.21%)	–	–
Total	205 (100%)	83 (40.49%)	122 (59.51%)	–	–

* *p < 0.05, statistically significant results*.

a *Gestation period (first trimester refers to the gestation period ranging from 1 to 12 weeks. Second trimester refers to the gestation period ranging from 13 to 28 weeks. Third trimester refers to the gestation period more than 28 weeks)*.

### The SCL-90 Inventory Score Distribution

All participants had varying degrees of psychological symptoms. The prevalence of psychological symptoms (a factor score ≥2) ranged from 6.83% (psychosis) to 17.56% (obsessive-compulsive). Of these symptoms, obsessive-compulsive, hostility, and phobic anxiety ranked as the highest, with a prevalence of 17.56, 16.10, and 14.63%, respectively. The prevalence of other psychological factors was ranked as follows: depression (13.17%), additional items (12.20%), somatization (10.73%), anxiety (10.73%), interpersonal sensitivity (9.27%), paranoid ideation (8.29%), and psychosis (6.83%) (Table 2).

**Table 2:** The distribution of SCL-90 score among all pregnant women in this study [*n* (%)].

**Factor**	**1 ≤ *i* < 2**	**2 ≤ *i* < 3**	**3 ≤ *i* < 4**	**4 ≤ *i* < 5**	**5**	***i* ≥ 2**
Somatization	183 (89.27%)	17 (8.29%)	5 (2.44%)	0 (0.00%)	0 (0.00%)	22 (10.73%)
Obsessive-compulsive	169 (82.44%)	31 (15.12%)	3 (1.46%)	2 (0.98%)	0 (0.00%)	36 (17.56%)
Interpersonal sensitivity	186 (90.73%)	15 (7.32%)	3 (1.46%)	1 (0.49%)	0 (0.00%)	19 (9.27%)
Depression	178 (86.83%)	23 (11.22%)	2 (0.98%)	2 (0.98%)	0 (0.00%)	27 (13.17%)
Anxiety	183 (89.27%)	18 (8.78%)	2 (0.98%)	2 (0.98%)	2 (0.98%)	24 (10.73%)
Hostility	172 (83.90%)	27 (13.17%)	5 (2.44%)	1 (0.49%)	0 (0.00%)	33 (16.10%)
Phobic anxiety	175 (85.37%)	24 (11.71%)	6 (2.93%)	0 (0.00%)	0 (0.00%)	3 (14.63%)
Paranoid ideation	188 (91.71%)	13 (6.34%)	3 (1.46%)	1 (0.49%)	0 (0.00%)	17 (8.29%)
Psychosis	191 (93.17%)	11 (5.37%)	3 (1.46%)	0 (0.00%)	0 (0.00%)	14 (6.83%)
Additional items	180 (87.80%)	20 (9.76%)	4 (1.95%)	1 (0.49%)	0 (0.00%)	25 (12.20%)

*i ≥ 2 means the occurrence of mental distress*.

### The Prevalence of Psychological Symptoms Between Pregnant HCWs and Pregnant Non-HCWs

**Figure 1:**
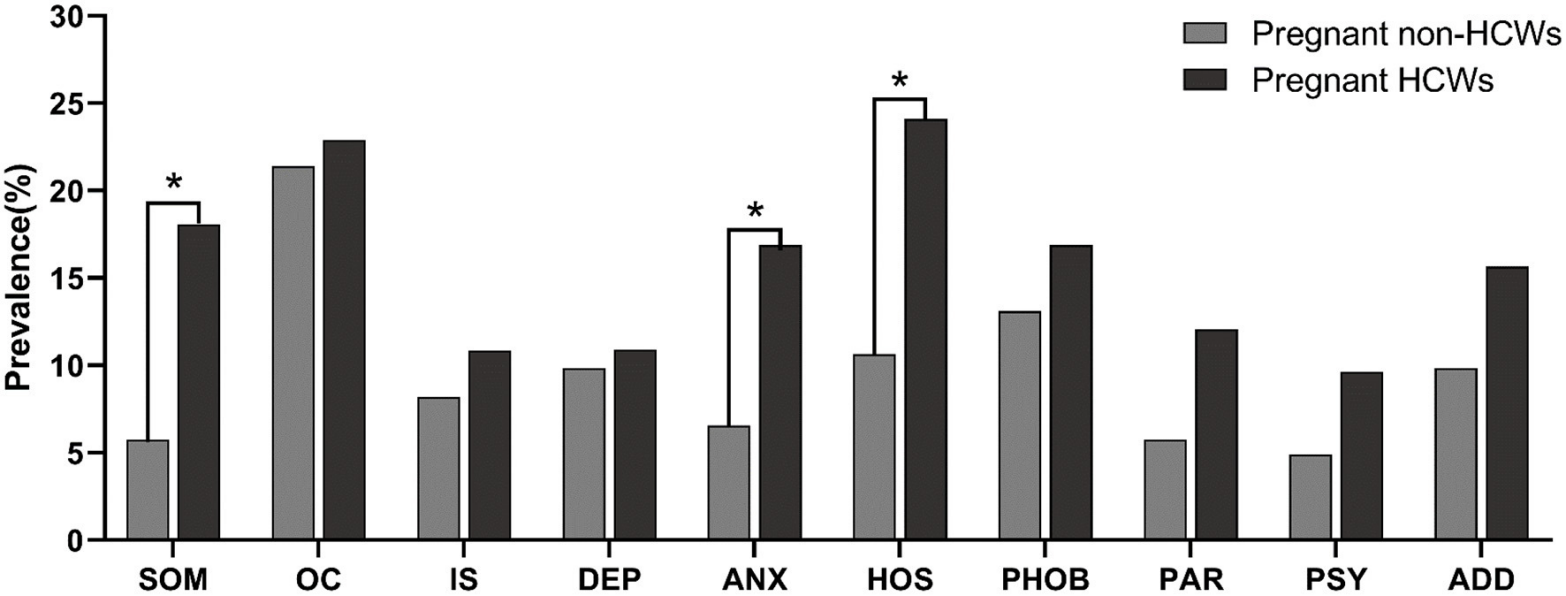
The prevalence of psychological symptoms (factor score ≥2) between pregnant HCWs and pregnant non-HCWs. ^*^p < 0.05, statistically significant results. SOM, somatization; OC, obsessive-compulsive symptoms; IS, interpersonal sensitivity; DEP, depression; ANX, anxiety; HOS, hostility; PHOB, phobic anxiety; PAR, paranoid ideation; PSY, psychosis; ADD, additional items.

Compared with pregnant non-HCWs, pregnant HCWs reported a higher prevalence of psychological symptoms in 10 factors of the SCL-90. Chi-square tests showed that pregnant HCWs had a significantly higher prevalence of somatization symptoms (18.07 vs. 5.74%), anxiety symptoms (16.87 vs. 6.56%), and hostility symptoms (24.10 vs. 10.66%) than pregnant non-HCWs (Table 3; Figure 1). After adjusting for the confounding variables of age, education, gestation period, mode of gestation, and number of fetuses, pregnant HCWs were still more likely to suffer from somatization symptoms [adjusted odds ratio (aOR) = 4.52, *p* = 0.006], anxiety symptoms (aOR = 3.54, *p* = 0.016), and hostility symptoms (aOR = 2.70, *p* = 0.027) than pregnant non-HCWs (Table 4). Further analysis revealed that “headaches” (*p* < 0.001), “faintness” (*p* = 0.007), “nausea or upset stomach” (*p* = 0.011), “hot or cold spells” (*p* = 0.003), and “heavy feelings in arms/legs” (*p* = 0.021) were the main causes of the significant difference in somatization symptoms between these two groups. Similarly, “heart pounding/racing” (*p* = 0.008) contributed to the between-group difference in anxiety symptoms between the two groups. “Urges to break things” (*p* < 0.001) and “shouting/throwing” (*p* = 0.010) contributed to the difference in hostility between the two groups (Supplementary Table S1).

**Table 3:** The prevalence of psychological symptoms between pregnant HCWs and pregnant non-HCWs.

**Variable**	**Pregnant HCWs (%)**	**Pregnant non-HCWs (%)**	**Statistic (χ^**2**^)**	***p*-Value**
Somatization ≥2	15 (18.07%)	7 (5.74%)	7.845	0.005^*^
Obsessive-compulsive symptoms ≥2	19 (22.89%)	17 (13.93%)	2.737	0.098
Interpersonal sensitivity ≥2	9 (10.84%)	10 (8.20%)	0.412	0.521
Depression ≥2	15 (18.07%)	12 (9.84%)	2.930	0.087
Anxiety ≥2	14 (16.87%)	8 (6.56%)	5.481	0.019^*^
Hostility ≥2	20 (24.10%)	13 (10.66%)	6.607	0.010^*^
Phobic anxiety ≥2	14 (16.87%)	16 (13.11%)	0.557	0.456
Paranoid ideation ≥2	10 (12.05%)	7 (5.74%)	2.587	0.108
Psychosis ≥2	8 (9.64%)	6 (4.92%)	1.730	0.188
Additional items ≥2	13 (15.66%)	12 (9.84%)	1.566	0.211

* *p < 0.05, statistically significant results*.

**Table 4:** Comparison of psychological symptoms among pregnant HCWs and pregnant non-HCWs.

**Variable**	**OR (95% CI)** ^**a**^ ^,^ ^**b**^	***p*-Value**	**aOR (95% CI)** ^**a**^ ^,^ ^**c**^	***p*-Value** ^**c**^
Somatization ≥2	3.63 (1.41, 9.33)	0.008^*^	4.52 (1.56, 13.16)	0.006^*^
Obsessive-compulsive symptoms ≥2	1.83 (0.89, 3.78)	0.101	2.11 (0.91, 4.91)	0.083
Interpersonal sensitivity ≥2	1.36 (0.53, 3.51)	0.522	1.42 (0.48, 4.20)	0.530
Depression ≥2	2.02 (0.89, 4.58)	0.091	1.86 (0.72, 4.83)	0.202
Anxiety ≥2	2.89 (1.15, 7.25)	0.024^*^	3.54 (1.26, 9.93)	0.016^*^
Hostility ≥2	2.66 (1.24, 5.71)	0.012^*^	2.70 (1.12, 6.51)	0.027^*^
Phobic anxiety ≥2	1.34 (0.62, 2.93)	0.457	1.73 (0.71, 4.21)	0.228
Paranoid ideation ≥2	2.25 (0.82, 6.18)	0.115	2.71 (0.87, 8.44)	0.086
Psychosis ≥2	2.06 (0.69, 6.18)	0.196	2.51 (0.73, 8.55)	0.142
Additional items ≥2	1.70 (0.74, 3.94)	0.214	1.96 (0.73, 5.25)	0.179

a *Pregnant non-HCWs were considered reference*.

b *Crude, no adjustment*.

c *After adjusted for age, education, gestation period, mode of gestation, and number of pregnant fetuses*.

* *p < 0.05, statistically significant results*.

### The Top Frequent SCL-90 Items (Score ≥2) in Pregnant HCWs and Pregnant Non-HCWs

Of the top frequent 20 items (score ≥2) of the SCL-90, different responses were found between the two groups. For pregnant HCWs, the unique symptoms were as follows: urges to break things, “poor appetite,” “repeating same actions,” “no interest in things,” “feeling blocked,” “heart pounding/racing”, and “difficulty making decisions.” For pregnant non-HCWs, the unique symptoms were as follows: “sleep that is restless or disturbed,” “overeating,” “headaches,” “others are to blame,” “feeling tense,” and “awakening in the early morning” (Supplementary Table S2). Further analysis demonstrated that there was little difference between these two groups in each dimension of the SCL-90. For example, in the “obsessive-compulsive” dimension, pregnant HCWs tended to report “having to check and double check what you do,” “having to repeat the same actions such as touching, counting, and washing,” and “feeling blocked in getting things done.” However, pregnant non-HCWs tended to report “unwanted thoughts or ideas that won't leave your head” and “worried about sloppiness or carelessness” in this dimension. The detailed information for differences in other dimensions is provided in Supplementary Table S3.

### Sensitivity Analysis

Three groups were divided as follows: group 1: not working pregnant non-HCWs; group 2: working pregnant non-HCWs; and group 3: pregnant HCWs. Compared with group 1, group 2 reported a similar prevalence of psychological symptoms in 10 factors of the SCL-90. However, group 3 reported a higher prevalence than the other two subgroups. Chi-square tests revealed significant differences in somatization and hostility symptoms between the three subgroups (Supplementary Table S4).

## Discussion

To the best of our knowledge, this is the first study to compare the prevalence of psychological symptoms between pregnant HCWs and pregnant non-HCWs during the early stage of the COVID-19 pandemic in China. Our study showed the following: (1) the prevalence of psychological symptoms among all pregnant women ranged from 6.83 to 17.56% and (2) symptoms of somatization, anxiety, and hostility in pregnant HCWs were significantly more severe than those in pregnantnon-HCWs.

In the current study, the prevalence of psychological symptoms among pregnant women ranged from 6.83 to 17.56%. These results are consistent with previous studies in China. Yu et al. reported that the rate of depressive symptoms among pregnant women in late pregnancy in Hengyang City was 9.2% (95% CI, 7.2–11.2%) (36). Zhou et al. showed that the detection rate of anxiety among pregnant women in Beijing was 6.8% during the COVID-19 epidemic (37). The variation in prevalence in other studies might be due to the different study locations and measurement tools. Lebel et al. (38), for example, found substantially elevated psychological symptoms in pregnant women (*n* = 1,987) in Canada, with 37% reporting clinically relevant symptoms of depression and 57% reporting clinically relevant symptoms of anxiety. Liu et al. (39) found that 36.4 and 22.7% of pregnant women (*n* = 1,123) in the USA reported clinically significant levels of depression and generalized anxiety, respectively. However, COVID-19 was emerging and quickly spreading in these countries during this period (40). Therefore, pregnant women in these countries (e.g., Canada and the USA) might have experienced higher level of depression and anxiety than those in China.

Our study revealed that pregnant HCWs were 4.52, 3.54, and 2.7 times more likely to report somatization, anxiety, and hostility symptoms than pregnant non-HCWs during the early stage of the COVID-19 pandemic. These results indicate that there are differences in psychological symptoms among pregnant HCWs due to their special work environment. A recent meta-analysis including 115 articles with 60,458 HCWs illustrated the high risk of developing mental health outcomes for HCWs related to coronavirus (SARS, Middle East Respiratory Syndrome, COVID-19) syndromes. The comprehensive results showed insomnia of 37.9%, psychological distress of 37.8%, burnout of 34.4%, anxiety features of 29%, depressive symptoms of 26.3%, and PTSD of 20.7% (41). Although few studies have evaluated the psychological impact of COVID-19 on pregnancy in HCWs, related studies on pregnancy have indicated that physiological and mechanical changes increase susceptibility to infections in general, which in turn might exacerbate mental health status (42). Given this, many experts have advised organizations and hospitals to provide more protective practices for pregnant HCWs (29). Further analysis demonstrated that behaviors such as headaches, nausea, or upset stomach, faintness, heart pounding/racing, and urge to break things contributed to the significant difference between the two groups. This could be because pregnant HCWs were more likely to have been exposed to a heavy workload, lack of rest, and fear of infection.

HCWs faced an overwhelming workload pressure and long working hours during the COVID-19 pandemic (18), which could have led to high levels of mental distress. The association between long-time work and somatization and anxiety among frontline HCWs has been shown previously (18). Clinical studies have found that, when under stress, the neuroendocrine network is regulated by the hypothalamic–pituitary–adrenal axis with an increasing level of the corticotropin-releasing factor and low level of cortisol, which leads to the continuous activation of the adrenergic pathway (43). As a result, negative emotions such as anxiety, irritability, hypersensitivity, and fear are more likely to occur.

Furthermore, a lack of rest might cause somatization symptoms in pregnant HCWs. With the daily surge in cases and the shortage of HCWs during the initial stage of the pandemic, working overtime and sleep deprivation might have become a normal phenomenon for pregnant HCWs. However, previous studies have indicated that a lack of rest can have a wide range of effects on physiological functions, such as cardiovascular, endocrine, immune system, and energy metabolism functions (44). Pregnant HCWs with these functions impaired would be more prone to suffer from a series of symptoms, such as headache, faintness, nausea or upset stomach, and heavy feelings in the arms and legs.

Finally, previous research has demonstrated that pregnant HCWs had higher risks of morbidity, mortality, and perinatal complications from infectious diseases (45), which indicates that they might bear more mental distress than general pregnant women. Fear of infection might be common. A relevant study reported that HCWs tend to show more intense fear and anxiety symptoms than the general population during outbreaks of infectious diseases (20). Although some HCWs do not come into direct clinical contact with infected patients, as do frontline HCWs, pregnant HCWs may still be afraid of infecting their family members with the disease due to commuting between the hospital and home. Additionally, pregnant HCWs might feel fear and anxiety about the possible threats to the health of their fetus in potential high-risk workplaces, such as fever clinics, emergency rooms, and pulmonary medicine departments (21). The occurrence of stressful events, such as witnessing infection or death of HCWs in person or on the news, might also lead to a psychological burden in pregnant HCWs. As of March 9, 2020, it has been reported that more than 3,000 HCWs in China have been infected with COVID-19 (24). The high infection rate and initially insufficient understanding of the virus might have made pregnant HCWs concerned about infection of themselves and their fetus.

The mother and fetus (even postnatally) are a dyad. Thus, pregnant HCWs' health could have large impacts on the health of their offspring. Previous studies have found that maternal psychological disorders were associated with the mental health and behaviors of their fetus and children (46, 47). For these reasons, it is necessary to provide more guidance and protective practices for pregnant women, especially those working in the healthcare system.

Our study has several limitations. First, we only detected psychological symptoms using self-report measures, without any careful diagnoses that followed structured clinical interviews by healthcare professionals. The respondents might have given inaccurate answers based on cultural and social expectations. Second, our study adopted a cross-sectional design, which prevents the investigation of causal relationships between related factors and psychological symptoms among pregnant women. Therefore, the results should be verified in future prospective cohort studies. Third, the participants with limited sample size were only from Chongqing, which limits the generalization of our findings to a wider population. Future studies should be conducted in a larger population with representative sampling methods in multiple sites.

## Conclusion

The current study indicated that compared with the pregnant non-HCWs, pregnant HCWs were more likely to report a higher prevalence of somatization, anxiety, and hostility symptoms. It is vital to implement targeted psychological interventions for pregnant women, especially for pregnant HCWs to cope with distress when facing the emerging infectious diseases.

## Data Availability Statement

The data presented in the paper are not readily available because ethics approvals do not permit the data to be made publicly available due to limitations of participant consent and concerns regarding potentials re-identifiability.

## Ethics Statement

The studies involving human participants were reviewed and approved by The Institutional Review Board of Jinshan Hospital, The First Affiliated Hospital of Chongqing Medical University. The patients/participants provided their written informed consent to participate in this study.

## Author Contributions

PX: has full access to all of the data in the study and takes responsibility for the integrity of the data, the accuracy of the data analysis, concept and design, critical revision of the manuscript for important intellectual content, and supervision. ML, NL, XC, XF, and RW: acquisition, analysis, or interpretation of data. ML, NL, and PX: drafting of the manuscript. ML and NL: statistical analysis. PX and ML: administrative, technical, or material support. All authors contributed to the article and approved the submitted version.

## Conflict of Interest

The authors declare that the research was conducted in the absence of any commercial or financial relationships that could be construed as a potential conflict of interest.

## Publisher's Note

All claims expressed in this article are solely those of the authors and do not necessarily represent those of their affiliated organizations, or those of the publisher, the editors and the reviewers. Any product that may be evaluated in this article, or claim that may be made by its manufacturer, is not guaranteed or endorsed by the publisher.

## Abbreviations

HCWs: healthcare workers
PTSD: posttraumatic stress disorder
SARS: severe acute respiratory syndrome
COVID-19: coronavirus disease 2019
SCL-90: symptom checklist-90
aOR: adjusted odds ratio.

## References

[1] Li Q Guan X Wu P Wang X Zhou L Tong Y. Early transmission dynamics in Wuhan, China, of novel coronavirus-infected pneumonia. N Engl J Med. (2020) 382:1199–207. 10.1056/NEJMoa2001316 31995857 PMC7121484

[2] Paules CI Marston HD Fauci AS. Coronavirus infections-more than just the common cold. JAMA. (2020) 323:707–8. 10.1001/jama.2020.0757 31971553

[3] World Health Organization. WHO Coronavirus Disease (COVID-19) Dashboard. (2020). Available online at: https://covid19.who.int/ (accessed Jan 15, 2021).

[4] Hossain MM Tasnim S Sultana A Faizah F Mazumder H Zou L. Epidemiology of mental health problems in COVID-19: a review. F1000Research. (2020) 9:636. 10.12688/f1000research.24457.1 33093946 PMC7549174

[5] Gao J Zheng P Jia Y Chen H Mao Y Chen S. Mental health problems and social media exposure during COVID-19 outbreak. PLoS ONE. (2020) 15:e0231924. 10.1371/journal.pone.0231924 32298385 PMC7162477

[6] Wang C Pan R Wan X Tan Y Xu L Ho CS. Immediate psychological responses and associated factors during the initial stage of the 2019 Coronavirus Disease (COVID-19) Epidemic among the general population in China. Int J Environ Res Public Health. (2020) 17:1729. 10.3390/ijerph17051729 32155789 PMC7084952

[7] Füzéki E Groneberg DA Banzer W. Physical activity during COVID-19 induced lockdown: recommendations. J Occup Med Toxicol. (2020) 15:25. 10.1186/s12995-020-00278-9 32817753 PMC7422663

[8] Shaukat N Ali DM Razzak J. Physical and mental health impacts of COVID-19 on healthcare workers: a scoping review. Int J Emerg Med. (2020) 13:1–8. 10.1186/s12245-020-00299-5 32689925 PMC7370263

[9] Salehi L Rahimzadeh M Molaei E Zaheri H Esmaelzadeh-Saeieh S. The relationship among fear and anxiety of COVID-19, pregnancy experience, and mental health disorder in pregnant women: a structural equation model. Brain Behav. 10:e01835. 10.1002/brb3.1835 32969190 PMC7536966

[10] Holditch-Davis D Santos H Levy J White-Traut R O'Shea TM Geraldo V. Patterns of psychological distress in mothers of preterm infants. Infant Behav Dev. (2015) 41:154–63. 10.1016/j.infbeh.2015.10.004 26495909 PMC4654120

[11] Liu X Chen M Wang Y Sun L Zhang J Shi Y. Prenatal anxiety and obstetric decisions among pregnant women in Wuhan and Chongqing during the COVID-19 outbreak: a cross-sectional study. BJOG. (2020) 127:1229–40. 10.1111/1471-0528.16381 32583536 PMC7362035

[12] Dong H Hu R Lu C Huang D Cui D Huang G. Investigation on the mental health status of pregnant women in China during the Pandemic of COVID-19. Arch Gynecol Obstetr. (2021) 303:463–9. 10.1007/s00404-020-05805-x 33009997 PMC7532741

[13] Zhang CJP Wu H He Z Chan NK Huang J Wang H. Psychobehavioral responses, post-traumatic stress and depression in pregnancy during the early phase of COVID-19 outbreak. Psychiatr Res Clin Pract. (2020) 3:46–54. 10.1176/appi.prcp.20200019 34172982 PMC7753825

[14] Zhang Y Ma ZF. Psychological responses and lifestyle changes among pregnant women with respect to the early stages of COVID-19 pandemic. Int J Soc Psychiatry. (2021) 67:344–50. 10.1177/0020764020952116 32815434 PMC8191160

[15] Rees S Channon S Waters CS. The impact of maternal prenatal and postnatal anxiety on children's emotional problems: a systematic review. Eur Child Adolesc Psychiatry. (2019) 28:257–80. 10.1007/s00787-018-1173-5 29948234 PMC6510846

[16] Liu X Kakade M Fuller CJ Fan B Fang Y Kong J. Depression after exposure to stressful events: lessons learned from the severe acute respiratory syndrome epidemic. Compr Psychiatry. (2012) 53:15–23. 10.1016/j.comppsych.2011.02.003 21489421 PMC3176950

[17] Maunder R Hunter J Vincent L Bennett J Peladeau N Leszcz M. The immediate psychological and occupational impact of the 2003 SARS outbreak in a teaching hospital. CMAJ. (2003) 168:1245–51. 12743065 PMC154178

[18] Zhou Y Wang W Sun Y Qian W Liu Z Wang R. The prevalence and risk factors of psychological disturbances of frontline medical staff in china under the COVID-19 epidemic: workload should be concerned. J Affect Disord. (2020) 277:510–4. 10.1016/j.jad.2020.08.059 32882508 PMC7448730

[19] Zhang WR Wang K Yin L Zhao WF Xue Q Peng M. Mental health and psychosocial problems of medical health workers during the COVID-19 epidemic in China. Psychother Psychosomat. (2020) 89:242–50. 10.1159/000507639 32272480 PMC7206349

[20] Tian F Li H Tian S Yang J Shao J Tian C. Psychological symptoms of ordinary Chinese citizens based on SCL-90 during the level I emergency response to COVID-19. Psychiatry Res. (2020) 288:112992. 10.1016/j.psychres.2020.112992 32302816 PMC7151383

[21] Chen Y Zhou H Zhou Y Zhou F. Prevalence of self-reported depression and anxiety among pediatric medical staff members during the COVID-19 outbreak in Guiyang, China. Psychiatry Res. (2020) 288:113005. 10.1016/j.psychres.2020.113005 32315886 PMC7160637

[22] Lu W Wang H Lin Y Li L. Psychological status of medical workforce during the COVID-19 pandemic: a cross-sectional study. Psychiatry Res. (2020) 288:112936. 10.1016/j.psychres.2020.112936 32276196 PMC7195354

[23] Luo M Guo L Yu M Jiang W Wang H. The psychological and mental impact of coronavirus disease 2019 (COVID-19) on medical staff and general public–a systematic review and meta-analysis. Psychiatry Res. (2020) 291:113190. 10.1016/j.psychres.2020.113190 32563745 PMC7276119

[24] Guangming Online. Beijing:Central Steering Group: Over 3,000 Medical Staff in Hubei Were Infected in the Early Stage of the Epidemic, Currently No Infection Reports Among Medical Aid Staff (2020). Available online at: https://politics.gmw.cn/2020–03/06/content_33626862.htm (accessed Jan 15, 2021).

[25] Cai H Tu B Ma J Chen L Fu L Jiang Y. Psychological impact and coping strategies of frontline medical staff in hunan between january and march 2020 during the outbreak of Coronavirus Disease 2019 (COVID-19) in Hubei, China. Med Sci Monit. (2020) 26:e924171-1–16. 10.12659/MSM.924171 32291383 PMC7177038

[26] Xiao H Zhang Y Kong D Li S Yang N. The effects of social support on sleep quality of medical staff treating patients with Coronavirus Disease 2019 (COVID-19) in january and february 2020 in China. Med Sci Monit. (2020) 26:e923549. 10.12659/MSM.923549 32132521 PMC7075079

[27] Xiao X Zhu X Fu S Hu Y Li X Xiao J. Psychological impact of healthcare workers in China during COVID-19 pneumonia epidemic: a multi-center cross-sectional survey investigation. J Affect Disord. (2020) 274:405–10. 10.1016/j.jad.2020.05.081 32663970 PMC7236675

[28] Chen M Zeng J Liu X Sun G Gao Y Liao J. Changes in physiology and immune system during pregnancy and coronavirus infection: a review. Eur J Obstet Gynecol Reprod Biol. (2020) 255:124–8. 10.1016/j.ejogrb.2020.10.035 33125977 PMC7566677

[29] Belingheri M Paladino ME Riva MA. Risk exposure to coronavirus disease 2019 in pregnant healthcare workers. J Occup Environ Med. (2020) 62:e370. 10.1097/JOM.0000000000001881 32730041 PMC7224594

[30] Australian Department of Health and Aotearoa New Zealand Ministry of Health. COVID-19: Guidance for Pregnant Healthcare Workers (2020). Available online at: https://www.racp.edu.au/docs/default-source/news-and-events/covid-19/covid-19-guidance-for-pregnant-healthcare-workers.pdf?sfvrsn=b22eec1a6 (accessed Jan 15, 2021).

[31] News I. Pregnant Healthcare Workers Advised to Minimise Their Patient-Fronting Duties During COVID-19 Pandemic InterventionalNews (2020). Available online at: https://interventionalnews.com/pregnant-healthcare-workers-covid/ (accessed jan 15, 2021).

[32] Yu Y Wan C Huebner ES Zhao X Zeng W Shang L. Psychometric properties of the symptom check list 90 (SCL-90) for Chinese undergraduate students. J. Mental Health. (2019) 28:213–9. 10.1080/09638237.2018.1521939 30465457

[33] Fan Y Wang B Zhai X Su Z. A test for the reliability and validity of SCL-90 in middle school student. Sichuan Mental Health. (2002) 15:132–5.

[34] Nunnally J editor. Psychometric Theory. 2nd ed. New York, NY: McGraw-Hill (1978).

[35] StataCorp L. Stata Statistical Software: Release 14. College Station, TX: StataCorp LP (2015).

[36] Yu Y Zhu X Xu H Hu Z Zhou W Zheng B. Prevalence of depression symptoms and its influencing factors among pregnant women in late pregnancy in urban areas of Hengyang City, Hunan Province, China: a cross-sectional study. BMJ Open. (2020) 10:e038511. 10.1136/bmjopen-2020-038511 32873680 PMC7467533

[37] Zhou Y Shi H Liu Z Peng S Wang R Qi L. The prevalence of psychiatric symptoms of pregnant and non-pregnant women during the COVID-19 epidemic. Transl Psychiatry. (2020) 10:319. 10.1038/s41398-020-01006-x 32950999 PMC7501755

[38] Lebel C MacKinnon A Bagshawe M Tomfohr-Madsen L Giesbrecht G. Elevated depression and anxiety symptoms among pregnant individuals during the COVID-19 pandemic. J Affect Disord. (2020) 277:5–13. 10.1016/j.jad.2020.07.126 32777604 PMC7395614

[39] Liu CH Erdei C Mittal L. Risk factors for depression, anxiety, and PTSD symptoms in perinatal women during the COVID-19 Pandemic. Psychiatry Res. (2020) 295:113552. 10.1016/j.psychres.2020.113552 33229122 PMC7904099

[40] AJMC Staff. A Timeline of COVID-19 Developments in 2020 (2020). Available online at: https://www.ajmc.com/view/a-timeline-of-covid19-developments-in-2020 (accessed Jan 15, 2021).

[41] Salazar de Pablo G Vaquerizo-Serrano J Catalan A Arango C Moreno C Ferre F. Impact of coronavirus syndromes on physical and mental health of health care workers: systematic review and meta-analysis. J Affect Disord. (2020) 275:48–57. 10.1016/j.jad.2020.06.022 32658823 PMC7314697

[42] Dashraath P Wong JLJ Lim MXK Lim LM Li S Biswas A. Coronavirus disease 2019 (COVID-19) pandemic and pregnancy. Am J Obstetr Gynecol. (2020) 222:521–31. 10.1016/j.ajog.2020.03.021 32217113 PMC7270569

[43] Sabban EL Serova LI Newman E Aisenberg N Akirav I. Changes in gene expression in the locus coeruleus-amygdala circuitry in inhibitory avoidance PTSD model. Cell Mol Neurobiol. (2018) 38:273–80. 10.1007/s10571-017-0548-3 28889197 PMC11481846

[44] Grandner MA Jackson NJ Pak VM Gehrman PR. Sleep disturbance is associated with cardiovascular and metabolic disorders. J Sleep Res. (2012) 21:427–33. 10.1111/j.1365-2869.2011.00990.x 22151079 PMC3703752

[45] Lynch L Spivak ES. The pregnant healthcare worker: fact and fiction. Curr Opin Infect Dis. (2015) 28:362–8. 10.1097/QCO.0000000000000180 26098508

[46] Stein A Pearson RM Goodman SH Rapa E Rahman A McCallum M. Effects of perinatal mental disorders on the fetus and child. Lancet. (2014) 384:1800–19. 10.1016/S0140-6736(14)61277-0 25455250

[47] Velders FP Dieleman G Henrichs J Jaddoe VW Hofman A Verhulst FC. Prenatal and postnatal psychological symptoms of parents and family functioning: the impact on child emotional and behavioural problems. Eur Child Adolesc Psychiatry. (2011) 20:341–50. 10.1007/s00787-011-0178-0 21523465 PMC3135831

