# Supplementary material for: The Prevalence of Psychological Symptoms in Pregnant Healthcare Workers (HCWs) and Pregnant Non-HCWs During the Early Stage of COVID-19 Pandemic in Chongqing, China
Source: Front Psychiatry. 2021 Aug 25;12:708698. doi: 10.3389/fpsyt.2021.708698 (PMC8424106; doi:10.3389/fpsyt.2021.708698)
Supplement: Supplementary file 1 [file Table_1.docx]

**Supplementary information**

| Table S1. The comparison of 90 items (score≥2) of SCL-90 among pregnant HCWs and pregnant non-HCWs [n(%)] | | | | |
| --- | --- | --- | --- | --- |
| **Items** | **Pregnant HCWs(%)** | **Pregnant non-HCWs(%)** | **Statistic (χ^2^)** | ***p*-Value** |
| **Somatization** |  |  |  |  |
| 1. Headaches | 9(10.84%) | 40(32.79%) | 13.076 | <0.001^*^ |
| 4. Faintness | 26(31.33%) | 19(15.57%) | 7.153 | 0.007^*^ |
| 12.Pains in heart/chest | 19(22.89%) | 24(19.67%) | 0.309 | 0.578 |
| 27.Pains in lower back | 52(62.65%) | 80(65.57%) | 0.184 | 0.668 |
| 40. Nausea or upset stomach | 42(50.60%) | 40(32.79%) | 6.532 | 0.011^*^ |
| 42. Soreness of your muscles | 38(45.78%) | 42(34.43%) | 2.677 | 0.102 |
| 48. Trouble getting your breath | 26(31.33%) | 33(27.05%) | 0.441 | 0.507 |
| 49. Hot or cold spells | 19(22.89%) | 10(8.20%) | 8.782 | 0.003^*^ |
| 52. Numbness or tingling in parts of your body | 16(19.28%) | 16(13.11%) | 1.424 | 0.233 |
| 53. A lump in your throat | 18(21.69%) | 22(18.03%) | 0.420 | 0.517 |
| 56. Feeling weak in parts of your body | 27(32.53%) | 28(22.95%) | 2.309 | 0.129 |
| 58. Heavy feelings in your arms/legs | 29(34.94%) | 25(20.49%) | 5.314 | 0.021^*^ |
| **Obsessive-compulsive symptoms** |  |  |  |  |
| 3. Unwanted thoughts, words, or ideas that won't  leave your mind | 30(36.14%) | 36(29.51%) | 0.997 | 0.318 |
| 9. Trouble remembering things | 54(69.23%) | 88(73.95%) | 0.521 | 0.470 |
| 10. Worried about sloppiness or carelessness | 27(32.53%) | 36(29.51%) | 0.212 | 0.645 |
| 28. Feeling blocked in getting things done | 31(37.35%) | 34(27.87%) | 2.050 | 0.152 |
| 38. Having to do things very slowly to insure  correctness | 22(26.51%) | 31(25.41%) | 0.031 | 0.860 |
| 45. Having to check and double check what you do | 34(40.96%) | 41(33.61%) | 1.153 | 0.283 |
| 46.Difficulty making decisions | 31(37.35%) | 37(30.33%) | 1.099 | 0.295 |
| 51. Your mind going blank | 26(31.33%) | 33(27.05%) | 0.441 | 0.507 |
| 55. Trouble concentrating | 27(32.53%) | 38(31.15%) | 0.044 | 0.835 |
| 65. Repeating same actions | 34(40.96%) | 33(27.05%) | 4.347 | 0.037 |
| **Interpersonal sensitivity** |  |  |  |  |
| 6. Feeling critical of others | 31(37.35%) | 47(38.52%) | 0.029 | 0.865 |
| 21. Feeling shy or uneasy with the opposite sex | 7(8.43%) | 9(7.38%) | 0.077 | 0.782 |
| 34.Your feelings being easily hurt | 28(33.73%) | 31(25.41%) | 1.670 | 0.196 |
| 36.Feeling others do not understand you or are  unsympathetic | 20(24.10%) | 32(26.23%) | 0.119 | 0.730 |
| 37. Feeling that people are unfriendly or dislike you | 16(19.28%) | 13(10.66%) | 3.023 | 0.082 |
| 41. Feeling inferior to others | 20(24.10%) | 26(21.31%) | 0.220 | 0.639 |
| 61. Feeling uneasy when people are watching or  talking about you | 22(26.51%) | 30(24.59%) | 0.096 | 0.757 |
| 69.Feeling very self-conscious with others | 12(14.46%) | 11(9.02%) | 1.468 | 0.226 |
| 73.Feeling uncomfortable about eating or drinking  in public | 17(20.48%) | 28(22.95%) | 0.176 | 0.675 |
| **Depression** |  |  |  |  |
| 5. Loss of sexual interest or pleasure | 31(37.35%) | 50(40.98%) | 0.273 | 0.601 |
| 14. Feeling low in energy or slowed down | 52(62.65%) | 73(59.84%) | 0.164 | 0.685 |
| 15. Thoughts of ending your life | 6(7.23%) | 4(3.28%) | 1.661 | 0.197 |
| 20. Crying easily | 39(46.99%) | 61(50.00%) | 0.179 | 0.672 |
| 22. Feeling of being trapped or caught | 9(10.84%) | 6(4.92%) | 2.556 | 0.110 |
| 26. Blaming yourself for things | 20(24.10%) | 19(15.57%) | 2.329 | 0.127 |
| 29. Feeling lonely | 22(26.51%) | 27(22.13%) | 0.520 | 0.471 |
| 30. Feeling blue | 27(32.53%) | 31(25.41%) | 1.234 | 0.267 |
| 31. Worrying too much about things | 30(36.14%) | 46(37.70%) | 0.052 | 0.820 |
| 1. No interest in things | 33(39.76%) | 25(20.49%) | 9.038 | 0.003^*^ |
| 54. Feeling hopeless about the future | 15(18.07%) | 17(13.93%) | 0.642 | 0.423 |
| 71. Feeling everything is an effort | 18(21.69%) | 23(18.85%) | 0.248 | 0.618 |
| 79. Feelings of worthlessness | 18(21.69%) | 15(12.30%) | 3.226 | 0.072 |
| **Anxiety** |  |  |  |  |
| 2. Nervousness or shakiness inside | 38(45.78%) | 54(44.26%) | 0.046 | 0.830 |
| 17. Trembling | 9(10.84%) | 5(4.10%) | 3.532 | 0.060 |
| 23. Suddenly scared for no reason | 15(18.07%) | 22(18.03%) | 0.0001 | 0.994 |
| 33. Feeling fearful | 25(30.12%) | 35(28.69%) | 0.049 | 0.825 |
| 39. Heart pounding/racing | 31(37.35%) | 25(20.49%) | 7.070 | 0.008^*^ |
| 57. Feeling tense | 29(34.94%) | 39(31.97%) | 0.197 | 0.657 |
| 72. Spells of terror/panic | 18(21.69%) | 22(18.03%) | 0.420 | 0.517 |
| 78. Feeling so restless you couldn't sit still | 15(18.07%) | 16(13.11%) | 0.946 | 0.331 |
| 80. Feeling that familiar things are strange or unreal | 13(15.66%) | 13(10.66%) | 1.118 | 0.290 |
| 86. Feeling pushed to get things done | 13(15.66%) | 32(26.23%) | 3.219 | 0.073 |
| **Hostility** |  |  |  |  |
| 11. Feeling easily annoyed or irritated | 51(61.45%) | 73(59.84%) | 0.054 | 0.817 |
| 24. Temper outbursts that you could not control | 39(46.99%) | 55(45.08%) | 0.072 | 0.788 |
| 63. Having urges to beat, injure, or harm someone | 14(16.87%) | 11(9.02%) | 2.843 | 0.092 |
| 67. Urges to break things | 43(51.81%) | 15(12.30%) | 38.011 | <0.001^*^ |
| 74. Getting into frequent arguments | 19(22.89%) | 18(14.75%) | 2.211 | 0.137 |
| 81. Shouting/ throwing | 14(16.87%) | 7(5.74%) | 6.655 | 0.010^*^ |
| **Phobic anxiety** |  |  |  |  |
| 13. Feeling afraid in open spaces or on the streets | 16(19.28%) | 15(12.30%) | 1.876 | 0.171 |
| 25. Feeling afraid to go out of your house alone | 21(25.30%) | 26(21.31%) | 0.445 | 0.505 |
| 47. Feeling afraid to travel on buses, subways, or  trains | 34(40.96%) | 53(43.44%) | 0.124 | 0.724 |
| 50. Having to avoid certain things, places, or  activities because they frighten you | 30(36.14%) | 30(24.59%) | 3.185 | 0.074 |
| 70. Feeling uneasy in crowds, such as shopping or at a movie | 18(21.69%) | 29(23.77%) | 0.121 | 0.728 |
| 75. Feeling nervous when you are left alone | 15(18.07%) | 14(11.48%) | 1.770 | 0.183 |
| 82. Feeling afraid you will faint in public | 7(8.43%) | 19(15.57%) | 2.274 | 0.132 |
| **Paranoid ideation** |  |  |  |  |
| 8. Feeling others are to blame for most of your  troubles | 25(30.12%) | 40(32.79%) | 0.162 | 0.687 |
| 18.Feeling that most people cannot be trusted | 14(16.87%) | 13(10.66%) | 1.667 | 0.197 |
| 43.Feeling that you are watched or talked about by  others | 9(10.84%) | 6(4.92%) | 2.557 | 0.110 |
| 68. Having ideas or beliefs that others do not share | 14(16.87%) | 9(7.38%) | 4.467 | 0.035^*^ |
| 76. Others not giving you proper credit for your  achievements | 14(16.87%) | 16(13.11%) | 0.557 | 0.456 |
| 83. Feeling that people will take advantage of you if  you let them | 13(15.66%) | 5(4.10%) | 8.247 | 0.004^*^ |
| **Psychosis** |  |  |  |  |
| 7. The idea that someone else can control your  body | 18(21.69%) | 18(14.75%) | 1.640 | 0.200 |
| 16. Hearing words that others do not hear | 39(46.99%) | 20(16.39%) | 22.56 | <0.001^*^ |
| 35. Other people being aware of your private  thoughts | 17(20.48%) | 21(17.21%) | 0.350 | 0.554 |
| 62. Having thoughts that are not your own | 16(19.28%) | 17(13.93%) | 1.044 | 0.307 |
| 77. Feeling lonely even when you are with people | 13(15.66%) | 17(13.93%) | 0.118 | 0.731 |
| 84. Having thoughts about sex that bother you a lot | 8(9.64%) | 15(12.30%) | 0.350 | 0.554 |
| 85. The idea that you should be punished for your  sins | 12(14.46%) | 19(15.57%) | 0.048 | 0.827 |
| 87. The idea that something serious is wrong with  your body | 30(36.14%) | 17(13.93%) | 13.789 | <0.001^*^ |
| 88. Never feeling close to another person | 23(27.71%) | 11(9.02%) | 12.478 | <0.001^*^ |
| 90. The idea that something is wrong with your mind | 7(8.43%) | 9(7.38%) | 0.077 | 0.782 |
| **Additional items** |  |  |  |  |
| 19. Poor appetite | 39(46.99%) | 20(16.39%) | 22.557 | <0.001^*^ |
| 44. Trouble falling asleep | 36(43.37%) | 59(48.36%) | 0.494 | 0.482 |
| 59. Thoughts of death or dying | 11(13.25%) | 7(5.74%) | 3.483 | 0.062 |
| 60. Overeating | 24(28.92%) | 55(45.08%) | 5.450 | 0.020^*^ |
| 64. Awakening in the early morning | 11(13.25%) | 39(31.97%) | 9.381 | 0.002^*^ |
| 66. Sleep that is restless or disturbed | 22(26.51%) | 69(56.56%) | 18.071 | <0.001^*^ |
| 89. Feelings of guilt | 14(16.87%) | 8(6.56%) | 5.481 | 0.019^*^ |
| **p* < 0.05, statistically significant results. | | | | |

| Table S2. The top 20 items (score≥2) of SCL-90 among pregnant HCWs and pregnant non-HCWs | | | | | |
| --- | --- | --- | --- | --- | --- |
| Pregnant HCWs | | | Pregnant Non-HCWs | | |
| Rank | Items | n(%) | Rank | Items | n(%) |
| 1 | Trouble remembering things | 54(65.06%) | 1 | Trouble remembering things | 88(72.13%) |
| 2 | Feeling low in energy or slowed down | 52(62.65%) | 2 | Pains in lower back | 80(65.57%) |
| 3 | Pains in lower back | 52(62.25%) | 3 | Feeling easily annoyed or irritated | 73(59.84%) |
| 4 | Feeling easily annoyed or irritated | 51(61.45%) | 4 | Feeling low in energy or slowed down | 73(59.84%) |
| 5 | **Urges to break things** | **43(51.81%)** | 5 | **Sleep that is restless or disturbed** | **69(56.56%)** |
| 6 | Nausea or upset stomach | 42(50.60%) | 6 | Crying easily | 61(50.00%) |
| 7 | **Poor appetite** | **39(46.99%)** | 7 | Trouble falling asleep | 59(48.36%) |
| 8 | Crying easily | 39(46.99%) | 8 | Temper outbursts that you could not control | 55(45.08%) |
| 9 | Temper outbursts that you could not control | 39(46.99%) | 9 | **Overeating** | **55(45.08%)** |
| 10 | Nervousness or shakiness inside | 38(45.78%) | 10 | Nervousness or shakiness inside | 54(44.26%) |
| 11 | Soreness of your muscles | 38(45.78%) | 11 | Feeling afraid to travel on buses, subways or trains | 53(43.44%) |
| 12 | Trouble falling asleep | 36(43.37%) | 12 | Loss of sexual interest or pleasure | 50(40.98%) |
| 13 | Having to check and double check what you do | 34(40.96%) | 13 | Feeling critical of others | 47(38.52%) |
| 14 | Feeling afraid to travel on buses, subways or trains | 34(40.96%) | 14 | Worrying too much | 46(37.70%) |
| 15 | **Repeating same actions** | **34(40.96%)** | 15 | Soreness of your muscles | 42(34.43%) |
| 16 | **No interest in things** | **33(39.76%)** | 16 | Having to check and double check what you do | 41(33.61%) |
| 17 | Loss of sexual interest or pleasure | 31(37.35%) | 17 | **Headaches** | **40(32.79%)** |
| 18 | Feeling critical of others | 31(37.35%) | 18 | **Others are to blame** | **40(32.79%)** |
| 19 | **Feeling blocked in getting things done** | **31(37.35%)** | 19 | Nausea or upset stomach | 40(32.79%) |
| 20 | **Heart pounding/racing** | **31(37.35%)** | 20 | **Feeling tense** | **39(31.97%)** |
| 20 | **Difficulty making decisions** | **31(37.35%)** | 20 | **Awakening in the early morning** | **39(31.97%)** |

| Table S3. The top 3 items (score≥2) of each dimension of SCL-90 among pregnant HCWs and pregnant non-HCWs | | | | | |
| --- | --- | --- | --- | --- | --- |
| Pregnant HCWs | | | Pregnant Non-HCWs | | |
| Rank | Items | n(%) | Rank | Items | n(%) |
|  | **Somatization** |  |  | **Somatization** |  |
| 1 | Pains in lower back | 52(62.65%) | 1 | Pains in lower back | 52(65.57%) |
| 2 | Nausea or upset stomach | 42(50.60%) | 2 | Soreness of your muscles | 42(34.43%) |
| 3 | Soreness of your muscles | 38(45.78%) | 3 | Headaches | 40(32.79%) |
|  |  |  | 3 | Nausea or upset stomach | 40(32.79%) |
|  | **Obsessive-Compulsive** |  |  | **Obsessive-Compulsive** |  |
| 1 | Trouble remembering things | 54(65.06%) | 1 | Trouble concentrating | 38(31.15%) |
| 2 | **Having to check and double check what you do** | **34(40.96%)** | 2 | Difficulty making decisions | 37(30.33%) |
| 2 | **Repeating same actions** | **34(40.96%)** | 3 | **Unwanted thoughts, words, or ideas that won't leave your mind** | **36(29.51%)** |
| 3 | **Feeling blocked in getting things done** | **31(37.35%)** | 3 | **Worried about sloppiness or carelessness** | **36(29.51%)** |
| 3 | Difficulty making decisions | 31(37.35%) |  |  |  |
|  | **Interpersonal Sensitivity** |  |  | **Interpersonal Sensitivity** |  |
| 1 | Feeling critical of others | 31(37.35%) | 1 | Feeling critical of others | 47(38.52%) |
| 2 | Your feelings being easily hurt | 28(33.73%) | 2 | **Feeling others do not understand you or are unsympathetic** | **32(26.23%)** |
| 3 | **Feeling uneasy when people are watching or talking about you** | **22(26.51%)** | 3 | Your feelings being easily hurt | 31(25.41%) |
|  | **Depression** |  |  | **Depression** |  |
| 1 | Feeling low in energy or slowed down | 52(62.65%) | 1 | Feeling low in energy or slowed down | 73(59.84%) |
| 2 | Crying easily | 39(46.99%) | 2 | Crying easily | 61(50.00%) |
| 3 | **No interest in things** | **33(39.76%)** | 3 | **Loss of sexual interest or pleasure** | **50(40.98%)** |
|  | **Anxiety** |  |  | **Anxiety** |  |
| 1 | Nervousness or shakiness inside | 38(45.78%) | 1 | Nervousness or shakiness inside | 54(44.26%) |
| 2 | **Heart pounding/racing** | **31(37.35%)** | 2 | Feeling tense | 39(31.97%) |
| 3 | Feeling tense | 29(34.94%) | 3 | **Feeling pushed to get things done** | **28(26.23%)** |
|  | **Hostility** |  |  | **Hostility** |  |
| 1 | Feeling easily annoyed or irritated | 51(61.45%) | 1 | Feeling easily annoyed or irritated | 73(59.84%) |
| 2 | **Urges to break things** | **43(51.81%)** | 2 | Temper outbursts that you could not control | 55(45.08%) |
| 3 | Temper outbursts that you could not control | 39(46.99%) | 3 | **Getting into frequent arguments** | **18(14.75%)** |
|  | **Phobic Anxiety** |  |  | **Phobic Anxiety** |  |
| 1 | Feeling afraid to travel on buses, subways or trains | 34(40.96%) | 1 | Feeling afraid to travel on buses, subways or trains | 53(43.44%) |
| 2 | Having to avoid certain things, places or activities | 30(36.14%) | 2 | Having to avoid certain things, places or activities | 30(24.59%) |
| 3 | **Feeling afraid to go out of your house alone** | **21(25.30%)** | 3 | **Feeling uneasy in crowds such as shopping or at a movie** | **29(23.77%)** |
|  | **Paranoid Ideation** |  |  | **Paranoid Ideation** |  |
| 1 | Feeling others are to blame for most of your troubles | 25(30.12%) | 1 | Feeling others are to blame for most of your troubles | 40(32.79%) |
| 2 | Feeling that most people cannot be trusted | 14(16.87%) | 2 | Others not giving you proper credit for your achievements | 16(13.11%) |
| 3 | Having ideas or beliefs that others do not share | 14(16.87%) | 3 | Feeling that most people cannot be trusted | 13(10.66%) |
| 3 | Others not giving you proper credit for your achievements | 14(16.87%) |  |  |  |
|  | **Psychoticism** |  |  | **Psychoticism** |  |
| 1 | The idea that something serious is wrong with your body | 30(36.14%) | 1 | **Other people being aware of your private thoughts** | **21(17.21%)** |
| 2 | **Never feeling close to another person** | **23(27.71%)** | 2 | The idea that you should be punished for your sins | 19(15.57%) |
| 3 | The idea that someone else can control your thoughts | 18(21.69%) | 3 | The idea that someone else can control your thoughts | 18(14.75%) |
|  | **Additional items** |  |  | **Additional items** |  |
| 1 | **Poor appetite** | **39(46.99%)** | 1 | **Sleep that is restless or disturbed** | **69(56.56%)** |
| 2 | Trouble falling asleep | 36(43.37%) | 2 | Overeating | 59(48.36%) |
| 3 | Overeating | 24(28.92%) | 3 | Trouble falling asleep | 55(45.08%) |

| Table S4. Sensitivity analysis (Comparison of psychological symptoms among three subgroups of pregnant women) | | | | | |
| --- | --- | --- | --- | --- | --- |
| Variable | Group 1(%)^a^ | Group 2(%)^b^ | Group 3(%)^c^ | Statistic(χ^2^) | *p-value* |
| Somatization≥2 | 1 (5.56%) | 6(5.77%) | 15(18.07%) | 7.845 | 0.020* |
| Obsessive-compulsive symptoms≥2 | 2(11.11%) | 15(14.42%) | 19(22.89%) | 2.854 | 0.240 |
| Interpersonal sensitivity≥2 | 1(5.56%) | 9(8.65%) | 9(10.84%) | 0.587 | 0.746 |
| Depression≥2 | 2(11.11%) | 10(9.62%) | 15(18.07%) | 2.960 | 0.228 |
| Anxiety≥2 | 1(5.56%) | 7(6.73%) | 14(16.87%) | 5.503 | 0.064 |
| Hostility≥2 | 3(16.67%) | 10(9.62%) | 20(24.10%) | 7.172 | 0.028* |
| Phobic anxiety≥2 | 2(11.11%) | 14(13.46%) | 14(16.87%) | 0.625 | 0.732 |
| Paranoid ideation≥2 | 1(5.56%) | 6(5.77%) | 10(12.05%) | 2.587 | 0.274 |
| Psychosis≥2 | 1(5.56%) | 5(4.81%) | 8(9.64%) | 1.743 | 0.418 |
| Additional items≥2 | 3(16.67%) | 9(8.65%) | 13(15.66%) | 2.486 | 0.289 |
| *Note:* ^a^Group 1: not working pregnant non-HCWs; ^b^Group 2: working pregnant non-HCWs; ^c^Group 3: pregnant HCWs; * *p* <0.05, statistically significant results | | | | | |
